# Supplementary material for: PBRM1 Cooperates with YTHDF2 to Control HIF-1α Protein Translation
Source: Cells. 2021 Jun 8;10(6):1425. doi: 10.3390/cells10061425 (PMC8228889; doi:10.3390/cells10061425)
Supplement: Supplementary file 1 [file cells-10-01425-s001.zip › Supplementary Information_Shmakova.pdf]

Supplementary Information_Shmakova et al.

Supplementary methods

Immunofluorescence

Cells were plated onto sterilised glass coverslips (VWR 19mm) in 35mm plates at a density of 1.2×10^5. Cells were then treated with siRNA transfection and hypoxia as earlier described. 72h after seeding the cells were fixed to coverslips with 3.7% paraformaldehyde (w/v) in PBS (pH 6.8) for 15 mins at 37 ℃ inside an incubator or hypoxia workstation, then washed with PBS and permeabilized in PBS-0.1% Triton X-100 (w/v) for 10 mins at room temperature. Cells were then washed with PBS and blocked with 1% donkey serum (v/v) in PBS 0.05% Tween (v/v) for 30 mins at room temperature. Cells were placed in a humidified chamber, incubated with 1:50 HIF-1α (610958, BD Biosciences) and YTHDF2 (Proteintech) antibodies diluted in 1% donkey serum (v/v) in PBS 0.05% Tween (v/v) overnight, washed 3x 5 mins in PBS, incubated for 1 hr with secondary antibody in 1% donkey serum (v/v) in PBS 0.05% Tween (v/v) and washed 3 x 5 mins in PBS. The nuclei were stained with 33.3 µM Hoechst (Sigma) for 2 mins. Coverslips were then washed in water and mounted onto slides (VWR) using mounting medium (H-1000; Vector labs) and sealed with nail varnish. Cells were analysed and images were acquired using a Zeiss Axio Observer Z.1 microscope as image stacks with Z-stacks of at least 20 0.1 µm images. Images were analysed using OMERO client software (Open Microscopy Environment) [1]. Average intensity was quantified for the whole cell and nucleus to generate a relative nuclear localisation of YTHDF2 for each cell.

Supplementary References:

1. Allan, C.; Burel, J.M.; Moore, J.; Blackburn, C.; Linkert, M.; Loynton, S.; Macdonald, D.; Moore, W.J.; Neves, C.; Patterson, A.; et al. OMERO: flexible, model-driven data management for experimental biology. *Nat Methods* **2012**, *9*, 245-253, doi:10.1038/nmeth.1896.

Supplementary Figures

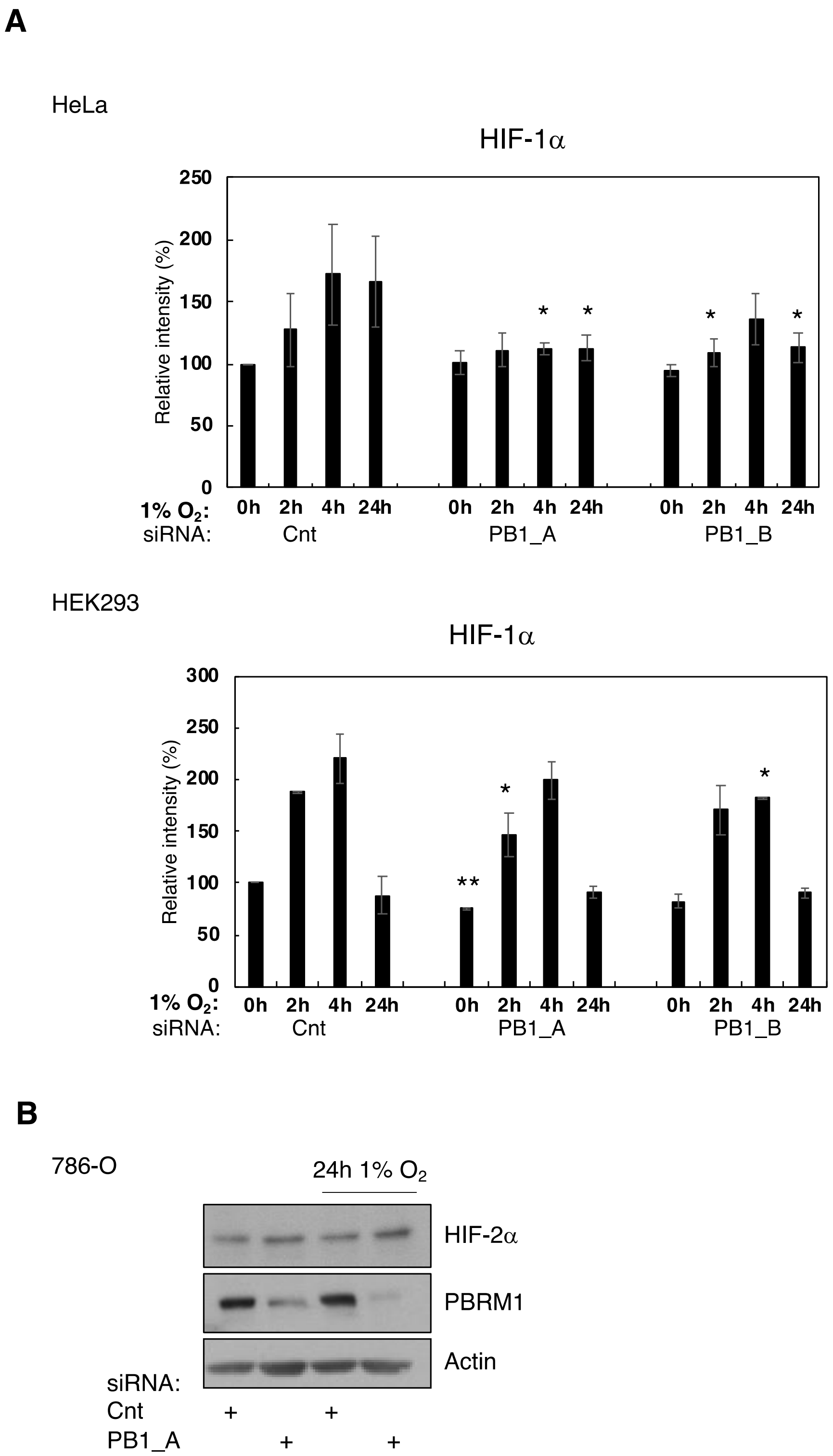

Figure S1. PBRM1 is required for hypoxia induced HIF-1α protein. (A) HeLa and HEK293 cells were transfected with control, or PBRM1 siRNAs for 48 hrs. The cells were exposed to to 1% O_2 for the indicated times and proteins were analysed by western blot. Intensities of HIF-1a protein normalised to Actin levels were quantified using Image J. Graph depicts mean and standard deviation of 3 replicates relative to control. Anova test was perfomed and significance indicated (* $p \leq 0.05$). **(B)** 786-O cells were transfected with control or PBRM1 siRNAs for 48 hrs. The cells were exposed to 1% O_2 for 24 hours prior to lysis and analysis by western blot.

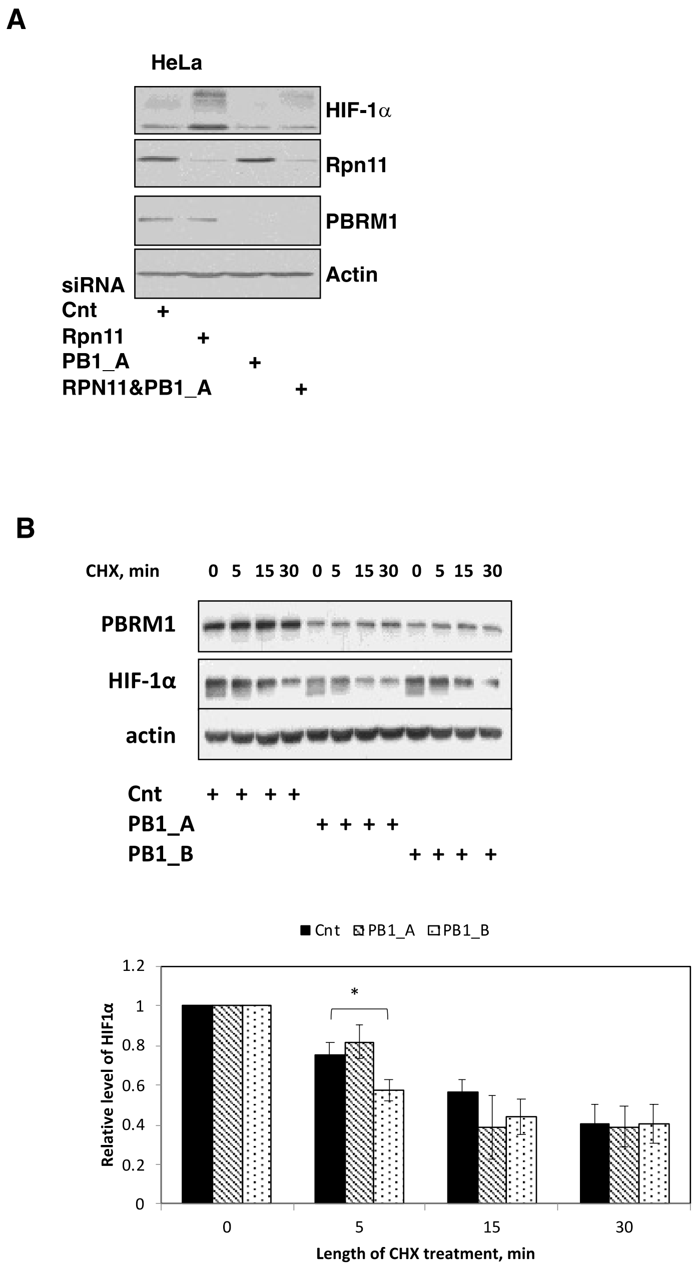

Figure S2. PBRM1 depletion does not affect HIF-1α protein stability. (A) HeLa cells were transfected with control, RPN11, PBRM1or a combination of these siRNAs for 48 hrs. The cells were exposed to MG132 for 3 hours and proteins were analysed by western blot. **(B)** HeLa cells were transfected with control or PBRM1 siRNAs for 48 hrs. The cells were exposed to 1% O_2 for 4 hours to stabilise HIF-1α protein, then treated with 0.1 mg/ml cycloheximide for indicated durations to inhibit translation. Protein turnover was monitored by western blot. The levels of HIF-1α were quantified from 3 replicates relative to no cycloheximide treatment. Means and standard deviation are plotted with Student's t-test significance indicated (* $p \leq 0.05$).

A

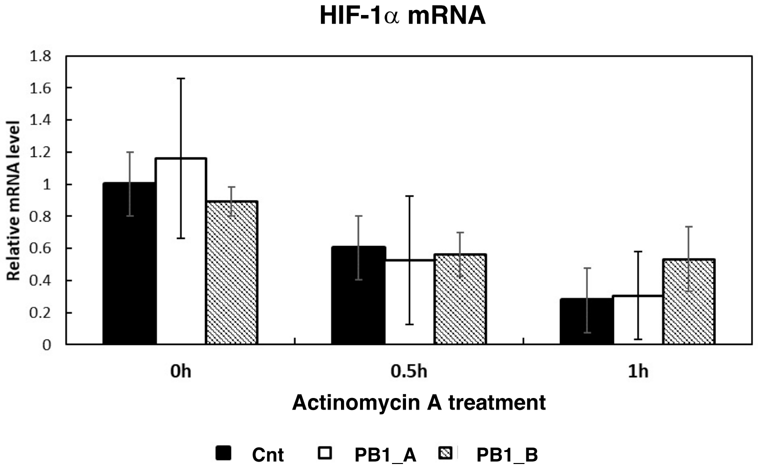

Figure S3. PBRM1 depletion does not affect HIF-1α RNA stability. (A) HeLa cells were transfected with control, RPN11, PBRM1or a combination of these siRNAs for 48 hrs. then treated with Actinomycin D for indicated durations. RNA was extracted and levels of HIF-1α were analysed by qPCR and normalised against actin and no Actinomycin D treatment. Means and standard deviation are plotted with Student's t-test significance investigated.

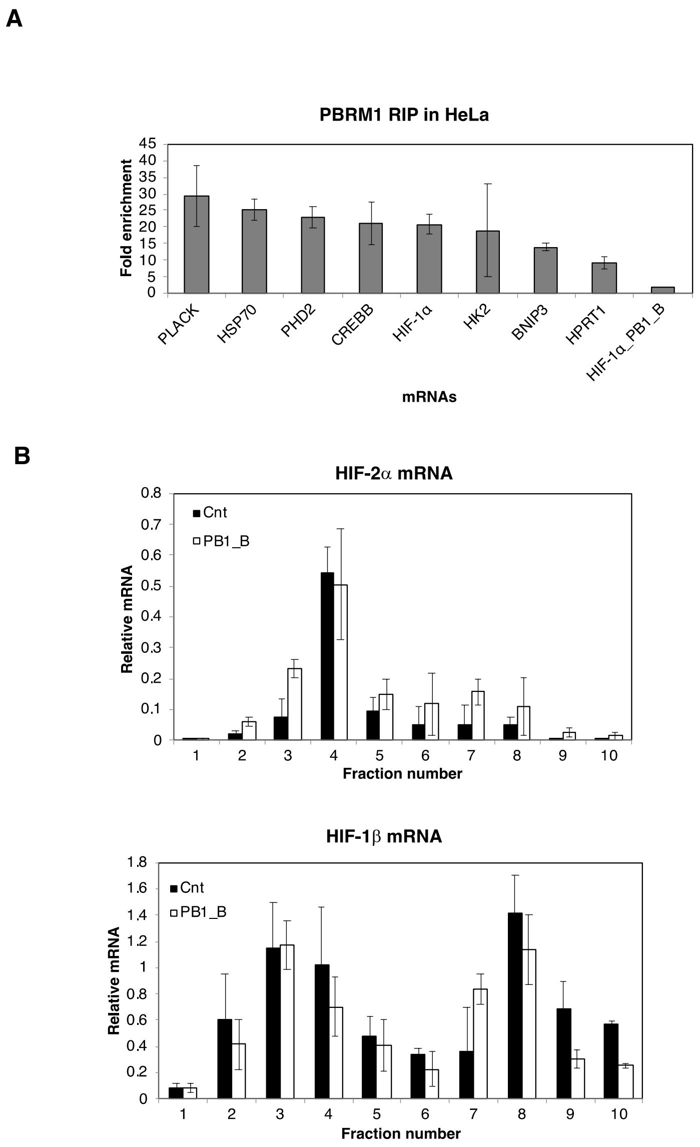

Figure S4. PBRM1 binds multiple RNAs but does not alter HIF-2α or HIF-1β translation. (A) HeLa cells were lysed and incubated with PBRM1 antibody coupled to magnetic beads. After washing any RNA present was converted to cDNA and analysed by qPCR using primers for the indicated hypoxia related genes. Levels were compared to an IgG control. Means and standard deviation are plotted. **(B)** HeLa cells were transfected with control or PBRM1 siRNA and lysed 48 hours later. Clarified HeLa cell lysate was added to a 10-50% sucrose gradient and centrifuged at 223,000 g then collected in fractions. RNA was extracted and levels of HIF-2α and HIF-1β were determined by qPCR for each fraction and reported relative to Actin levels. The means and standard deviations are plotted with Anova significance indicated.

A

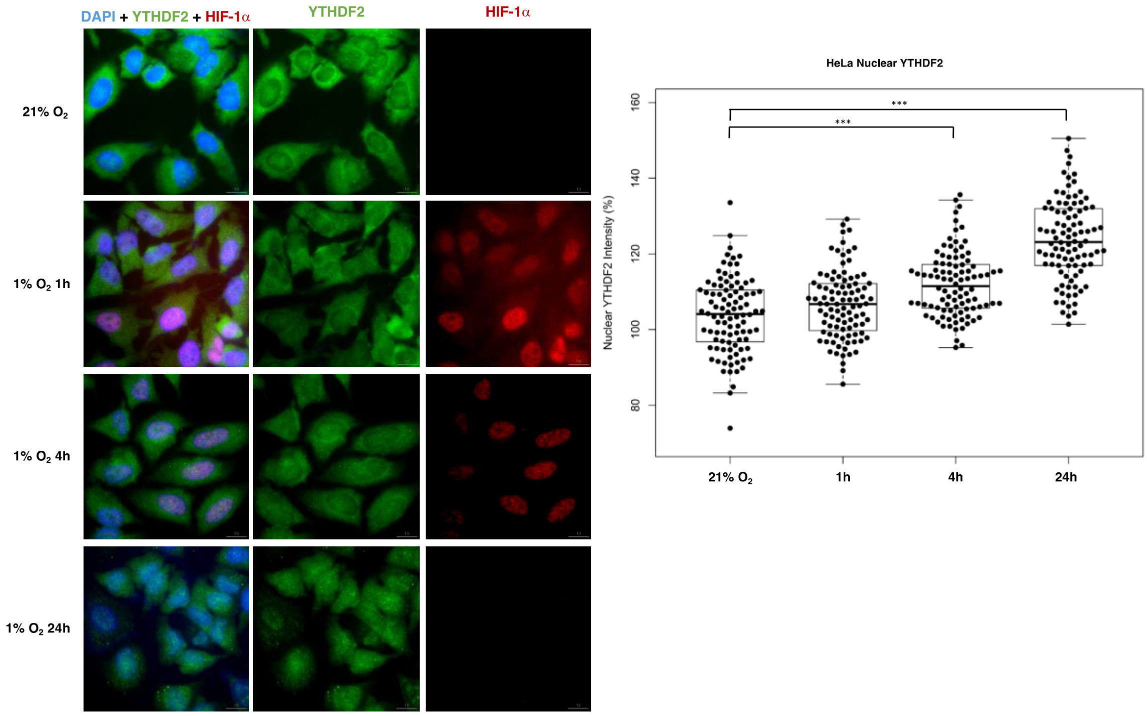

Figure S5. Hypoxia induces YTHDF2 nuclear accumulation. (A) HeLa cells were exposed to the indicated times of 1% O_2 prior to fixation and immunofluorescence analysis. Bar indicates 10 µm. Levels of nuclear YTHDF2 were quantified using the ROI tool in OMERO. Graph shots dot plots and Anova significance was investigated (*** p≤0.001).
